# Empowering Sustainability: Floating Solar Photovoltaic Systems in Agriculture for Reduced Costs Carbon Emissions and Evaporation

**DOI:** 10.1002/gch2.202300321

**Published:** 2024-06-17

**Authors:** Mahnaz Gümrükçüoğlu Yiğit, Bevin Akçadağ

**Affiliations:** ^1^ Department of Environmental Engineering Engineering Faculty Sakarya University Sakarya 54050 Türkiye

**Keywords:** agricultural sustainability, carbon emission reduction, floating solar photovoltaic, renewable energy investments, water conservation

## Abstract

This study assesses the impact of implementing a floating solar photovoltaic system (FSPV) on the Turgutlu irrigation pond in Sakarya, Turkey, aiming to reduce energy expenses in agricultural irrigation and promote sustainability in farming. Two scenarios are developed to evaluate the FSPV, focusing on CO_2_ emissions mitigation, energy generation potential, evaporation reduction, conservation of terrestrial land, effects on agricultural production, decreased reliance on fossil fuels, and associated costs and return on investment (ROI). In the first scenario, the FSPV is expected to generate 7168 MWh of energy, preventing the emission of 4520 tons of carbon, and reducing annual evaporation by 6686 m^3^. In the second scenario, the FSPV's energy output is estimated at 99 MWh, preventing 64.2 tons of carbon emissions, and reducing annual evaporation by 94.4 m^3^. These findings provide valuable insights at the regional level, presenting a compelling case study for potential replication in other irrigated agricultural regions.

## Graphical abstract

The impact of implementing floating solar photovoltaic systems (FSPV) on agricultural sustainability in Turkey's Sakarya region is explored. Assessing CO_2_ emissions reduction, energy generation, and evaporation mitigation, two scenarios reveal significant potential for FSPV adoption. Findings suggest replicable strategies for enhancing sustainability in irrigated agricultural areas.

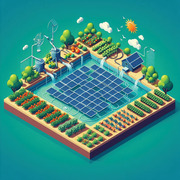

## 1 Introduction

In today's era marked by the climate crisis, it is evident that the increasing energy, food, and water consumption due to a rapidly growing population will lead to significant ecological and economic stress in the future. Utilizing renewable energy potential becomes crucial for ensuring energy security, preserving water resources, mitigating climate change, and promoting sustainable development. Solar energy, as an abundant and environmentally sustainable source, emerges as the optimal choice to meet the escalating energy demand compared to finite fossil fuel resources.

The latest approach to this issue involves the integration of photovoltaics with water sources, giving rise to a new applied solar technology known as FSPV systems. These systems harness solar energy directly, converting it into electricity without harming the environment, thus serving as a compelling solution.^[^ ^1^ ^]^

Unlike ground‐mounted solar panels, FSPVs are gaining prominence as a competitive option due to their efficiency and sustainability. Placed on water surfaces, they offer a more sustainable alternative by not occupying usable land and are cost‐competitive compared to ground‐mounted solar power plants. FSPV systems implemented on agricultural irrigation ponds reduces the carbon footprint in agriculture, preventing land occupation, saving water and energy by minimizing evaporation, lowering production inputs for farmers, and supporting sustainable agriculture.^[^ ^2^ ^]^

The agriculture sector, with its high overall costs and carbon footprint, particularly in water and energy consumption, can benefit significantly from the adoption of solar energy. Implementing solar power systems in agricultural irrigation presents an ecological and economic solution, making irrigation systems more sustainable and improving the efficient management of energy and water resources across the entire agriculture sector.

Agricultural irrigation ponds prove to be the most suitable areas for FSPVs. These systems enhance water use efficiency by preventing transport and evaporation losses, leading to up to 90% improvement with more efficient irrigation methods.^[^ ^3^ ^]^ FSPVs offer advantages such as reduced land leveling costs, shorter installation times, and higher electricity generation compared to fixed solar panels.^[^ ^4^ ^]^ Moreover, FSPV installed on natural lakes limit the purposes of use such as lake ecosystem, fish farming, tourism, and drinking water.^[^ ^5^ ^]^

When FSPVs are not constructed on terrestrial land, land leveling costs decrease, and there is no competition with sectors where land use is crucial, such as agriculture, mining, and tourism. Considering the adverse effects of expansive solar panel installations on land and their contribution to drought, FSPVs are becoming an increasingly competitive option.

Furthermore, FSPVs benefit from the cooling properties of surrounding water and the absence of dust to prevent the overheating of solar panels, resulting in higher electrical performance efficiency. A floating photovoltaic system producing 10 MW has 10.2% more production capacity compared to a land‐based PV system.^[^ ^6^ ^]^

Considering the anticipated intensification of hydrological, agricultural, and ecological droughts in the Mediterranean basin due to climate change, effective water resource management becomes increasingly crucial.^[^ ^7^ ^]^ FSPV systems can contribute to a 60% reduction in annual evaporation by minimizing the contact of the water surface with the sun.^[^ ^8^ ^]^ Various studies have shown that these systems decrease evaporation by reducing exposure to sunlight and wind effects on the water surface.^[^ ^9^, ^10^, ^11^ ^]^ Research conducted in Australia indicated that a floating PV system could result in at least 15000 m^3^ water savings per MWp.^[^ ^12^ ^]^ Estimates for lakes and dams in Turkey suggest potential water savings of over 20%.^[^ ^13^ ^]^ Furthermore, protecting water from solar radiation can improve water quality by reducing photosynthesis and weed growth. Particularly in arid and semi‐arid climates, reducing evaporation losses from the water surface can lead to more efficient management of stored water in ponds.

Compared to traditional fixed photovoltaic (FV) applications, FSPV systems incur higher initial investment, operational, and maintenance costs. The lifespan of floating systems is shorter than ground‐mounted FV systems due to increased corrosion in metallic structures.^[^ ^14^, ^15^, ^16^, ^17^ ^]^

The development of floating solar PV gained momentum in 2007 in Japan after a research‐oriented 20 kW FSPV project.^[^ ^18^ ^]^ Since then, it has proliferated in countries such as the United States, Italy, Spain, France, South Korea, and Singapore.^[^ ^19^ ^]^ China has completed the world's largest FSPV project, the Dezhou Dingzhuang FSPV project, with an expected global installed capacity of 62 GW by 2030.^[^ ^20^, ^21^ ^]^

Several global studies have investigated FSPV systems. One such study in 2019, entitled “Floating Solar Power Plant for Sustainable Development: A Techno‐economic Analysis,” explored the feasibility of a 10 MW FSPV plant, revealing that it generates 10.2% more power than a land‐based system.^[^ ^6^ ^]^ Another study focused on FSPV's technical potential in irrigation ponds and water infrastructures, finding that it prevents the occupation of 12 km^2^ of land and saves 8.8 ×106 m^3^ per year of evaporating water.^[^ ^22^ ^]^ In a 2020 study, the electricity generation and water‐saving capacity of a 1.14 MW FSPV system covering 30% of a reservoir were analyzed, demonstrating energy production, water savings, and CO_2_ reduction benefits.^[^ ^23^ ^]^ Additionally, a study on FSPV's technical potential in artificial water bodies found that covering 1% of eligible areas could generate energy equivalent to about 12.5% of national electricity generation.^[^ ^24^ ^]^ A study in Almeria, Spain, estimated a 17% water loss due to evaporation in agricultural reservoirs, with predicted losses of 60 hm3 in the Segura Basin, signifying over 8% of the current water source for irrigation.^[^ ^25^ ^]^ Sahu et al. examined the environmental and economic aspects of FSPV,^[^ ^16^ ^]^ and Trapani & Santafe presented flexible FSPV projects.^[^ ^25^ ^]^

Turkey, influenced by the Mediterranean climate, is deemed vulnerable to the impacts of climate change. In the projections of the effects of the climate crisis, Turkey has been evaluated among the regions in the world where an increase in water stress is expected. The projected decrease in the annual flows of rivers and precipitation after 2050 will also lead to a decrease in agricultural production. To align with the anticipated global warming trends, mitigating carbon emissions by transitioning from fossil fuels to renewable energy sources becomes imperative across various sectors. In adherence to international agreements, Turkey has identified key objectives, including the advancement of climate‐smart agriculture technologies, promoting sustainability in agriculture through decreased reliance on inputs, widespread adoption of renewable energy sources, ensuring energy efficiency, optimizing water utilization, and reducing dependency on fossil fuels.^[^ ^26^, ^27^ ^]^

In 2021, 53.3% of Turkey's energy demand was met from renewable energy sources, while 46.7% relied on fossil fuels. Within renewable energy sources, hydropower constituted the largest share at 31.55%.^[^ ^28^ ^]^ The installed capacity based on solar energy in Turkey is 9425.4 MW, representing a 9.08% share among other installed power sources.^[^ ^29^ ^]^ The country's dependence on imported sources for energy, especially in meeting natural gas demands, poses a significant challenge to its sustainable development goals, with 98.5% of natural gas needs being imported.^[^ ^30^ ^]^ Consequently, increasing the installed capacity based on solar energy emerges as one of the most effective solutions for promoting sustainable energy in Turkey.

Given Turkey's long sunshine duration (an average of 2358 h per year) and considerable solar radiation of 1527 kWh m^−2^‐year due to its geographical location, the country holds immense potential for solar energy utilization, allowing for significant reductions in carbon emissions resulting from energy consumption.^[^ ^30^ ^]^ Turkey's theoretical potential is advantageous compared to many European countries, including Germany, France, and Italy.^[^ ^31^ ^]^ In 2022, the contribution of solar‐generated installed electricity capacity to the total installed capacity increased by 1.25%.^[^ ^29^ ^]^

A significant portion of agricultural lands in Turkey relies on canals, streams, or ponds managed by the Directorate General for State Hydraulic Works (DSI) for water, employing pressurized irrigation systems. With a total of 690 agricultural irrigation ponds, there appears to be substantial FSPV potential across Turkey.^[^ ^32^ ^]^ The first FSPV installation in Turkey took place in Mersin in 2014, and additional installations have been implemented on Istanbul's Kucukcekmece and Buyukcekmece lakes.^[^ ^33^ ^]^

The aim of this study was to serve as a foundational work on the development of FSPV energy systems, specifically focusing on the connection between climate change, agricultural carbon footprint, water scarcity, and sustainable agriculture and energy transformation. The study, centered around the FSPV to be constructed on the Turgutlu irrigation pond in Sakarya, determines the energy required for agricultural irrigation, the reduction in CO_2_ emissions, the decrease in evaporation, the potential number of trees that can be planted on gained terrestrial land, the carbon sequestration capacity, and the amount of fossil fuel that can be saved as a ton of oil equivalent (TOE). The project's cost and its economic impact on the region and country are evaluated, providing a case study demonstrating the feasibility of implementing FSPV on artificial water resources in regions practicing irrigated agriculture, especially in semi‐arid climates.

The study is expected to contribute to sustainable energy production in the agricultural sector by providing data on energy generation, evaporation, greenhouse gas emissions, water scarcity, carbon footprint, and energy relationships. It is designed for implementation in regions practicing irrigated agriculture, utilizing artificial water resources, and emphasizing their beneficial outcomes. The obtained results will, in turn, offer evidence‐based guidance for policymakers and financial institutions in implementing similar applications.

In this study, we delineate the objectives and importance, accompanied by a thorough review of the existing global literature on FSPV systems. The specific attributes of the study area and the detailed methodology employed for the assessment of FSPV potential, quantification of CO_2_ reductions, and estimation of evaporation/water savings under different surface coverage scenarios, as well as the consequential effects on land use, agricultural production, and system costs, are comprehensively expounded. Moreover, these findings are meticulously compared with existing literature to contextualize and validate the study's outcomes.

## 2 Experimental Section

### 2.1 Sakarya City and Turgutlu Pond

Sakarya, the location of the study area, Turgutlu pond, is situated in the Marmara Region of Turkey (29‐31°E, 41‐40°N). The total population of the province is 1042649, with approximately 17% of the employed population working in the agricultural sector. The land in the province is 34% plains, 44% plateaus, and 22% mountains. The annual temperature average is 14.2 °C, the precipitation average is 800 mm, and the relative humidity average is 72%.^[^ ^34^ ^]^ In Sakarya, the sunshine duration is 2358 hours per year. The average evaporation amount is 837.9 mm per year while and the annual evaporation amount is 22288 m^3^ per year.^[^ ^30^ ^]^

Agriculture holds a primary position in the provincial economy and plays a significant role in Turkey's overall agriculture and economy. In Sakarya, total agricultural lands make up 49.4% of the provincial surface area, surpassing the national average. Among these agricultural lands, 30.5% is categorized as absolute agricultural land, and 44.5% is designated as planted agricultural land.^[^ ^34^ ^]^ The province's main agricultural products include hazelnuts, corn, quince, wheat, sugar beets, sunflowers, potatoes, various vegetables, and tobacco. Within these lands, 40% are dedicated to the cultivation of fruits that specifically require irrigation, while 41% are allocated for field crops and vegetable cultivation.

The total irrigable area in the province is 930 000 decares (1 decare = 1000 m^2^), of which 201920 decares are currently under irrigation (**Table** **1**).^[^ ^35^ ^]^

**Table 1:** Irrigability status of agricultural lands and water resources.

Irrigability status of Sakarya agricultural lands (x 1000 m^2^)	
Irrigable area	930000
Economically irrigable area	728080
Area opened for irrigation	201.920
State irrigation from above‐ground sources	166920
Public irrigation from above‐ground sources	21000
Public irrigation from underground sources	14000

Throughout the province, since pump systems are used in the irrigation of the agricultural areas in the plains, the energy requirement for irrigation is high. Accordingly, there are many agricultural irrigation ponds available for irrigation purposes. Of these agricultural irrigation ponds, irrigation is performed by using electrical pump systems in 52.8% and by taking advantage of the slope without using energy in 47.1%.^[^ ^36^ ^]^

**Figure 1:**
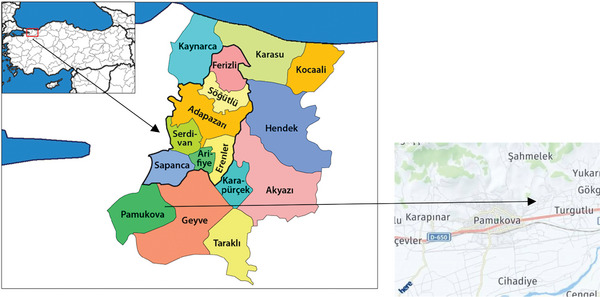
Map of Turgutlu pond location.

The Turgutlu Pond which of Project area has been being built on Degirmendere in Pamukova district, the agricultural center of Sakarya Province, for irrigation of 980 da agricultural land (**Figure** **1**).

The irrigation from this pond aims to enhance agricultural sustainability and contribute to the national economy. Technical information about the pond is provided in **Table** **2**.^[^ ^32^ ^]^

**Table 2:** Turgutlu irrigation pond technical data.

Turgutlu Pond	
Total pond area	38000 m^2^
Status	Under construction
Min water level	167.15 m
Average water level	175.00 m
Max water level	176.52 m
Active pond volume	0.220 hm^3^
Height above Thalweg	26.70 m
Height from foundation	34.50 m
Pump Q (350 l/s = 0.4*3600)	1260 m^3^ h^−1^
Pump power	350 kW
Total run time	283,16
Annual energy requirement	99.106 kW
Irrigation water use	3640,66 m^3^ ha^−1^
Total Irrigation Water Requirement	356.784,68 m^3^ ha^−1^

### 2.2 FSPV System Design

The main objective of this study is not to create a new method but to demonstrate a new application showing that well‐known and widely used FSPV systems are a very effective tool in terms of sustainability. By taking into account the installations of FSPV systems in areas with similar geographical conditions, the study plans to establish the lowest‐cost system suitable for the current conditions. Calculations have been carried out using data from the Turgutlu reservoir. Such integrated applications are very important, especially in developing countries and semi‐arid regions, to protect soil and water resources, ensure food security, and achieve sustainability by reducing the carbon footprint and costs in agriculture. Therefore, the study method is designed in this direction.

**Figure 2:**
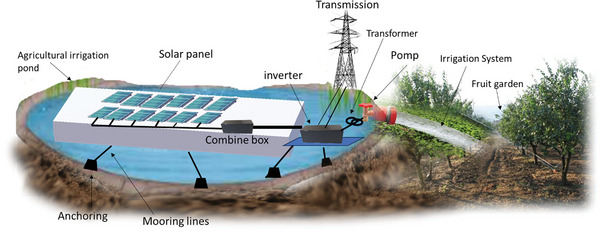
FSPV system design.

FSPV system, which is currently under installation on the pond, encompasses components akin to those in a standard solar photovoltaic (SPV) system. The first segment involves a floating system comprising a series of floating plastic modules that cover the water surface, intercepting solar radiation for power generation. The second part consists of the mooring system responsible for adjusting ripples. The third part includes the PV equipment mounted on the floating system, and the last segment is represented by the underwater cable facilitating the transfer of generated energy to the PV system.^[^ ^2^ ^]^ Materials for the platform include hollow plastic materials, flexible rubber or MDPE straps allowing buoys to move relative to each other for adapting to different water levels, ropes used to connect the outer modules of the floating cover to the edges of the reservoir, and reinforced concrete piles anchoring the floating cover. Designed to completely cover the lower surface, the platforms virtually minimize solar radiation transmission underneath. The size and shape of the floating module are crucial technical design requirements. The design of the FSPV system depends on the type of PV panel, the angle of the panels, the meteorological conditions of the site, and the support system.^[^ ^37^ ^]^ All panels will be placed in the same direction and at the same angle. To enhance electrical performance, the panel tilt angles will be less than 10 degrees, with a preference for minimizing the angle due to lower costs. Assuming a panel has a production capacity of 300 kW, irregular shapes conforming to the topography of the terrain are deemed suitable for the design of the floating platform and the reservoir's geometry (**Figure** **2**).

Studies conducted near Sakarya's latitude and southern orientation indicate that tilt angles of 10° or lower provide the minimum initial cost for producing 1 kWh of energy.^[^ ^38^ ^]^

### 2.3 Carbon Emissions

In this study, unlike existing studies, one of the main objectives is to reduce the carbon footprint of agricultural irrigation to support farmers and agriculture for sustainability. In Turkey, 36% of the energy is obtained from coal‐fired power plants. The energy required for agricultural irrigation is also supplied by these plants. Therefore, the carbon footprint in this study has been determined by comparing it with the footprint created by energy obtained from burning coal. The resulting carbon savings have been determined separately for two different scenarios. Since the carbon emissions are calculated using publicly available electricity consumption data, only qualitative uncertainty analysis has been performed.

FSPV installation offers a key advantage in reducing CO_2_ emissions, and the emissions saved with FSPV are higher compared to ground‐mounted SPVs.^[^ ^6^ ^]^ CO_2_ reductions were calculated for each scenario and compared with coal usage. In Turkey, CO_2_ emissions from the production of 1 kW are 0.6482 CO_2_ per kWh. The annual energy to be produced by FSPV was multiplied by this value and the carbon emission amount was found. Since this energy will not be obtained by using fossil fuels, it has been accepted that all emissions will be prevented. A comparison was made considering that if the energy obtained from FSPV were to be produced by burning fossil fuels, the CO_2_ emissions, which would occur from the combustion of fossil fuels, would be entirely eliminated/prevented with carbon emission‐free FSPV.

The prevention of CO_2_ and the associated savings can be achieved when the energy produced by FSPV is compared to the scenario where it is obtained using fossil fuels. The value of the prevented CO_2_ was calculated using this data, which is crucial for obtaining carbon certificates. Obtaining carbon certificates supports local renewable energy production and encourages the participation of local farmers, reducing their expenses. This is a significant step, especially in countries like Turkey, where the economy relies on agriculture, promoting the sustainability of agriculture.

### 2.4 Evaporation

To determine the reduction in pond surface evaporation with FSPV, the first step involved identifying the annual average evaporation in Sakarya. This was achieved by calculating the mean of the annual evaporation values from three districts with meteorological stations. Specifically, these values were 776.2 mm in Adapazari (the central district), 991.8 mm in Geyve district, and 745.7 mm in Sapanca district.^[^ ^39^ ^]^ The average of these district values, totaling 837.9 mm per year, was used in the computation of pond surface evaporation. The calculation considered a constant coefficient of 0.7, denoted as the pan constant by the meteorological station, to determine the total evaporation volume. To minimize the data error margin in evaporation calculations, the average of data collected from three different measurement stations was used.

The water savings resulting from the closure of 50% of the reservoir and the reduction in evaporation were estimated based on the potential value from previous studies.^[^ ^13^ ^]^ Approximately, this corresponds to about 10% of the reservoir's storage capacity.

### 2.5 Costs

The application of FSPV systems is not widespread, leading to uncertainties in installation and operating expenses. In the cost calculations, the error margin can be high due to variations in regional pricing of the parameters. However, because there are parameters such as labor costs that can show significant differences even within different regions of the same country, this error margin has not been calculated in the study. Regional parameters such as water level fluctuations, temperature, and wind play a crucial role in the installation and operation of these systems, making cost data from existing studies invaluable. The installation of floating solar power plants is reported to be 20–25% more expensive compared to ground‐mounted PV systems. The higher installation cost is attributed to specialized components such as floating structures, anchoring systems, underwater cables, and stabilizing vaults.^[^ ^6^, ^12^ ^]^

For cost calculations, equipment prices and labor wages necessary for establishing the floating SPV were determined by averaging current market prices.^[^ ^40^ ^]^ Parameters considered in this calculation included expropriation cost, PV panel price ($97.76 per piece), solar inverter, electrical cable equipment, required labor for installation, carrier system, and associated labor. The cost of installing the FSPV in half of the pond area in Scenario 1 was computed to be $2483200, while the cost for Scenario 2, based on the energy required for irrigation, amounted to $127384.

## 3 Results

In this study, calculations were conducted based on two distinct criteria to determine the optimal ecological and economic conditions for implementing the FSPV system.

The first criterion involved planning to cover only half of the total pond area with solar panels, and subsequent calculations were carried out accordingly. The aim here was to assess the impact and feasibility of implementing the FSPV system when covering a specific portion of the pond surface.

The second criterion focused on calculating the energy required to pump the necessary irrigation water based on the size of the agricultural areas to be irrigated. The system was designed to generate the energy needed for pumping water for irrigation, aligning the FSPV capacity with the irrigation demands of the agricultural fields. This criterion aimed to address the specific energy requirements related to irrigation practices in agriculture.

The fundamental values considered for the calculations related to the installation and impacts of floating solar panels on the pond surface are outlined as follows:

– Emission factor for energy generated from solar or wind = 0.6482 kg CO_2_ per kWh^[^ ^41^ ^]^
– Emission factor for energy generated from Lignite = 1.279 ton CO_2_ per MWh.^[^ ^42^ ^]^
– Electricity unit price for agricultural irrigation = 1.6 TL
– 1 Ton Equivalent Oil (TEO) = 11,640 kWh
– Proportion of evaporation prevented by the FSPV = 60%
– Each panel power = 400 W = 0.4 kW h^−1^
– Each panel size = 2 m^2^
– Space between panels = 0.5 m
– One tree assumed to be planted on an area of 3 m^2^ in the terrestrial area gained with the establishment of the SPV on the pond.
– 1 quince tree = 150–200 kg of quince yields

In the first scenario, aiming to safeguard water quality and quantity, 50% of the Turgutlu irrigation pond's total surface area, amounting to 38000 m^2^, will be allocated for the installation of the FSPV system. Consequently, the overall area designated for FSPV installation will be 19000 m^2^, with each solar panel covering 2 m^2^. Considering a planned spacing of 50 cm between the panels, a total of 7600 panels will be strategically positioned on the pond surface, enabling year‐round energy generation. The surplus energy generated outside the irrigation period will be supplied to the electricity grid through collaboration with the utility company.

To determine the energy output from a standard panel, the average efficiency of both the most efficient and least efficient panels available in the market under current conditions was calculated. Throughout the system's operational period, a performance loss of 25% is assumed due to factors such as module efficiency degradation, dirt on panels, temperature effects, inverter losses, system breakdowns, etc. Considering that it will be the first FSPV system installed on an agricultural irrigation pond, there is a possibility of higher efficiency loss due to operational errors. Therefore, the system's efficiency is conservatively estimated to be below the 75% global performance ratio, assumed to be around 60–70%.^[^ ^10^ ^]^ With an assumed energy production of 0.4 kWh per panel, the system is projected to generate a total annual energy output of 7168,320 kWh (7,168 MWh).

The annual CO_2_ emissions from the system, which is projected to produce 7,168 MWh of electricity annually, are estimated to be 4647 tons. In comparison, if the same amount of energy were generated by burning coal, it would result in approximately 9167.8 tons of CO_2_ emissions. Consequently, the implementation of this system is anticipated to contribute to a reduction of approximately 4520 tons of CO_2_ emissions per year. The intention is to leverage this emission reduction in the international voluntary carbon trade.^[^ ^41^ ^]^

Solar PV systems are classified as a clean energy source during operation since they do not emit greenhouse gases. However, there are pre‐operational greenhouse gas emissions associated with the production, transportation, and installation stages of PV systems. The Life Cycle Emissions of the system encompass all CO_2_ emissions arising from the construction and operation of FSPV installation. As noted in various studies, the amount of CO_2_ saved is often considered negligible compared to the emissions produced. Examples from previous similar studies evaluating the embedded carbon value associated with activities such as the production and transportation of materials that make up the FSPV have been considered. Santafe and co‐workers estimated embedded greenhouse gas emissions for FSPV systems as 137.73 kgCO_2_‐eq m^−2^.^[^ ^10^ ^]^ Another study considered it as 46 gCO_2_ per kWh.^[^ ^37^ ^]^ In the qualitative uncertainty analysis, the error margin for CO_2_ emissions in emission factors related to electricity production and fuel combustion, based on numerous measurement‐based estimates, ranges between 10% and 30%. This error margin has been accepted in the calculations for the scenarios created in the study.^[^ ^41^ ^]^

By covering half of the pond area with panels, the FSPV system is expected to prevent annual water evaporation of 6686 m^3^. Agricultural irrigation reservoirs, being fully integrated into the irrigation and water supply network of geographical regions with profitable agricultural activities, ensure the use of land in food production and the preservation of productive land by providing irrigation. The gained terrestrial area resulting from the FSPV installation on the pond will allow the planting of 6333 trees. Assuming the average carbon sequestration capacity of a tree to be 22 kg CO_2_ per year, this translates to sequestering 151 992 kg CO_2_ per year.^[^ ^45^ ^]^ Additionally, preventing the burning of fossil fuel equivalent to 615.8 tons of oil will contribute to environmental conservation. The system is projected to recoup its installation cost of $2483200 within a short period of 3.5 years (**Table** **3**). The trees to be planted in the newly acquired terrestrial area will be fruit trees, with quince being the primary product in the region. An average quince tree yields 150–200 kg of quince, valued at around $0.29 per kilogram. Planting 6333 quince trees is expected to generate a minimum income of $289855.

**Table 3:** Data calculated for the floating SPP of Turgutlu pond.

	First scenario floating SPP to be built using half of the pond area	Second scenario floating SPP to be installed according to the amount of energy used for irrigation
FSPV area	19000 m^2^	262.5 m^2^
Annual generation	7168,320 kWh ≌7168 MWh	99106 kWh ≌ 99 MWh
Number of panels	7600	105
Installment cost	$2483200	$127384
Prevented CO_2_ emission	4647 ton CO_2_ per year	64.2 ton CO_2_ per year
Evaporation amount prevented by FSPV	6686 m^3^ per year	94.4 m^3^ per year
Number of trees to be able to be planted in the area gained	6333	88
Carbon amounts that will be sequestered by the trees	151,992 kg CO_2_ per year	2112 kg CO_2_ per year
TEO of the energy procured	615.8 TEO per year	8.5 TEO/ per ear
Profit that will be gained by the energy generated by FSPV	$664887.6 per year	$9192 $ per year
Pay‐off period of the system	3.7 year	13.8 year
Proportion of meeting the energy need for irrigation	< %100	%100

In Scenario 1, the cost of installing the FSPV on half of the pond area is $2483200. The energy unit price to be sold to the government (calculated at 1.6 TL per kWh, and converted to dollars at the exchange rate of 17.25 dollars per TL) has been taken into account. Additionally, the burning of fossil fuel equivalent to 615.8 tons of oil will be prevented. The system is projected to recoup its installation cost of $2483200 within a short period of 3.5 years (Table 3).

In Scenario 1, where the state's specified irrigation fee of 1.6 TRY per kWh is not paid, the FSPV system can generate an annual profit of $664887.6. The cost to produce the required 99 106 kWh of electricity for annual irrigation will be $9192.4 per year. After subtracting this energy cost, the remaining energy will be sold to the electricity company, resulting in an annual profit of $655695.2, with a cost of $17.25 per plate number.

In Scenario 2, the FSPV is designed to meet the energy needs for irrigating a 980 da area from the pond using a pump, with 105 panels installed to harness the annual sunshine duration of 2358 h. The system will generate a total of 99,106 kWh ≈ 99 MWh of energy annually. This will lead to a reduction of 64.2 tons per year in CO_2_ emissions compared to energy systems relying on fossil fuels. The average carbon sequestration amount of 88 trees planted in the terrestrial area gained by installing the SPV on the pond will be 2,112 kg CO_2_ per year. If these trees are quince trees, the average income that can be obtained will be $4000. The avoidance of fossil fuel use for energy generation will prevent the burning of fossil fuel equivalent to 8.5 TEO annually. It was calculated that the system will be able to recoup the investment cost of $127384 in 13.8 years (Table 3).

## 4 Discussion and Assessment

The study focuses on reducing the carbon footprint to align with climate adaptation policies and conserving water resources. This is achieved through the utilization of solar energy in agricultural irrigation, which helps to decrease associated expenses, prevent encroachment on agricultural lands, and support sustainability in agriculture. Energy costs, especially for irrigation in agricultural activities, are notably high. Consequently, limited irrigation leads to inefficiency and reduced crop yield, ultimately causing issues in the country's economy alongside food price increases. This is particularly crucial for developing economies that heavily rely on agriculture. Sharing the results with decision‐makers and ensuring the inclusion of FSPV use in agricultural policies are the primary outcomes of this study.

Technically, numerous studies have been conducted on energy generation from solar panels, providing insights into research and practical applications in this field. Leveraging the results of these studies, this project aims to elucidate the contributions of FSPVs to the agricultural sector and highlight their impact on atmospheric and ecosystem balance by explaining the intended goals in the application phase. Therefore, data related to the production of solar panels, such as materials, installation angles, and geometry, have been determined by drawing upon studies conducted in regions with similar climatic and geographical conditions.

In Turkey, studies on SPV are limited and still in the design phase. The initial study focusing on using energy for agricultural irrigation in Turkey, and similar examples worldwide, are scarce. SPV are considered as novel and adaptable systems, particularly in agricultural ecosystems and less developed semi‐arid regions. They aim to reduce agricultural irrigation costs and carbon footprint while preserving fertile lands and water resources. Moreover, the goal is to demonstrate that these systems do not impose a burden on national economies in terms of costs. Solar panels and SES are highlighted as effective tools in this regard.

In the study, upon comparing the two scenarios, it becomes evident that the first scenario prevented approximately 70 times more CO_2_ emissions. The first scenario also exhibits a significant reduction in evaporation, contributing to water resource conservation. Conversely, the second scenario has a lower installation cost but requires a longer time to recover the investment.

Implementing the FSPV system, covering 50% of the pond area, is evidently a prudent investment, despite its initial high installation cost. The system brings forth various benefits, including the reduction of carbon emissions, mitigation of water surface evaporation, generation of additional income, and achieving a short pay‐off period.

In Turkey, with a total of 690 agricultural irrigation ponds, the potential for FSPV is substantial. If an FSPV system producing 7168 MWh per year of electrical energy is established in each of these ponds, it can contribute approximately 4945 920 MWh per year of electricity, constituting 1.5% of Turkey's total annual energy consumption. Moreover, the implementation of the system in 690 ponds across Turkey could prevent around 3.2 Mt of CO_2_ emissions annually.

The significance of this project lies in its pioneering establishment on an agricultural irrigation pond, becoming the first of its kind in Turkey. While previous studies primarily emphasized the value of utilizing solar energy for power generation, this study goes beyond and highlights its dual impact: not only reducing carbon emissions from agriculture but also serving as a crucial energy source for agricultural irrigation. This dual benefit, especially in the context of Turkey's agricultural reliance, holds the potential to set a precedent for other nations engaged in irrigated agriculture. The study will be an important step in demonstrating to decision‐makers that small‐scale systems, requiring minimal investment and occupying small areas, can contribute to agriculture and support farmers, especially in areas with highly profitable and productive agricultural lands. Moreover, the choice of artificial irrigation ponds as the installation site ensures the preservation of natural lake ecosystems, as discussed earlier. The findings indicating that SPVs on lakes can mitigate evaporation align with results from prior studies, as seen in Bangladesh,^[^ ^43^ ^]^ and the observed reduction in the carbon footprint is consistent with findings in earlier research.^[^ ^44^ ^]^ These aspects collectively contribute to the uniqueness and broader relevance of the project.

The construction of FSPVs on ponds ensures the protection of agricultural and forest lands. In large‐scale projects, the higher cost of FSPV is offset by the expenses associated with acquiring and expropriating unused land. In cases where land value is substantial, FSPV systems exhibit better economic performance compared to ground‐mounted PV systems. Moreover, our study emphasizes the potential for cultivating agricultural products in areas where SPVs are not installed on land. The acquired land in the study area is crucial due to the high fertility of the soils for agriculture. The revenue generated from agricultural products will contribute to the faster return on investment for the FSPV system. One of the unique aspects of this study is the calculation of sample areas and products as an indicator of the economic contribution to agriculture and farmers, demonstrating how cultivating crops in fertile lands will contribute to agriculture and provide economic support to farmers. The elimination of terrestrial land occupation, as indicated in the study, aligns with findings from previous research.^[^ ^22^ ^]^ This outcome also plays a role in maintaining the sustainability of urban planning and controlling land prices in urban areas.^[^ ^6^ ^]^ The calculated installation cost and return‐on‐investment period for the FSPV system align with results from various studies, considering its energy production capacity.^[^ ^44^ ^]^

Recently, carbon emissions in Turkey have experienced rapid growth, with high energy expenses being a significant component in various sectors like agriculture, industry, transportation, and heating. Approximately 31.43% of this energy is sourced from coal‐fired power plants, contributing to increased carbon emissions. Given that 45% of these coal‐fired plants rely on imported coal, rising coal prices further escalate electricity generation costs. In 2021, the average cost for existing thermal power plants operating with imported coal reached $73 per MWh, while the new solar plant's cost was calculated at $51.9.^[^ ^46^ ^]^ Consequently, newly established solar power plants offer more cost‐effective electricity generation compared to existing imported coal power plants. Utilizing solar energy for agricultural irrigation not only aids in reducing agricultural input costs, positively impacting the economy but also helps mitigate carbon emissions, addressing climate change concerns. Thus, the integration of solar energy into the agricultural sector presents numerous ecological and economic advantages. Currently, 40% of the world's food production is derived from the irrigated 18% portion of agricultural lands, underscoring the critical importance of sustainability in irrigated agriculture.^[^ ^46^ ^]^

Particularly in arid and semi‐arid regions such as the Mediterranean, there exists significant potential for the establishment of floating solar panel electricity generation facilities to improve water and energy balances.^[^ ^47^ ^]^

## 5 Conclusion

In contemporary times, agriculture and food production face significant challenges stemming from climate change and heightened carbon emissions. Addressing these challenges requires the development and implementation of innovative approaches and systems to ensure sustainability in the agricultural sector. FSPV systems present a, solution by optimizing land use efficiency, mitigating carbon emissions from energy generation, reducing evaporation losses, lessening dependence on foreign energy sources, and minimizing ecological and economic costs. In this study, the primary objective was to elucidate the ecological and economic advantages and the critical importance of sustainability in agriculture offered by the Turgutlu Floating Solar Photovoltaic project.

In conclusion, the Turgutlu FSPV project presents a pivotal opportunity for Sakarya province to contribute significantly to the national economy through renewable energy investments. By addressing the resource‐intensive nature of modern agriculture, the FSPV system is poised to protect fertile agricultural lands, enhance environmental sustainability, and bolster regional agriculture and economy. The project's potential to reduce carbon emissions, optimize energy costs, and mitigate evaporation makes it a crucial component in Turkey's pursuit of clean energy, zero carbon emissions, and sustainable growth. As a national and international exemplar, the FSPV system stands to set a precedent for similar initiatives, positioning itself as a reference for the sector and fostering advancements in renewable energy and sustainability on a broader scale.

## Conflict of Interest

The authors declare no conflict of interest.

## Data Availability

The data that support the findings of this study are available from the corresponding author upon reasonable request.
